# The Flavonoid Glabridin Induces OCT4 to Enhance Osteogenetic Potential in Mesenchymal Stem Cells

**DOI:** 10.1155/2017/6921703

**Published:** 2017-11-14

**Authors:** June Seok Heo, Seung Gwan Lee, Hyun Ok Kim

**Affiliations:** ^1^Department of Integrated Biomedical and Life Sciences, College of Health Science, Korea University, Seoul 02841, Republic of Korea; ^2^Cell Therapy Center, Severance Hospital, Seoul 03722, Republic of Korea; ^3^Department of Health and Environmental Science, College of Health Science, Korea University, Seoul 02841, Republic of Korea; ^4^Department of Laboratory Medicine, Yonsei University College of Medicine, Seoul 03722, Republic of Korea

## Abstract

Mesenchymal stem cells (MSCs) are a promising tool for studying intractable diseases. Unfortunately, MSCs can easily undergo cellular senescence during *in vitro* expansion by losing stemness. The aim of this study was to improve the stemness and differentiation of MSCs by using glabridin, a natural flavonoid. Assessments of cell viability, cell proliferation, *β*-galactosidase activity, differentiation, and gene expression by reverse transcription PCR were subsequently performed in the absence or presence of glabridin. Glabridin enhanced the self-renewal capacity of MSCs, as indicated by the upregulation of the *OCT4* gene. In addition, it resulted in an increase in the osteogenic differentiation potential by inducing the expression of osteogenesis-related genes such as *DLX5* and *RUNX2*. We confirmed that glabridin improved the osteogenesis of MSCs with a significant elevation in the expression of *OSTEOCALCIN* and *OSTEOPONTIN* genes. Taken together, these results suggest that glabridin enhances osteogenic differentiation of MSCs with induction of the *OCT4* gene; thus, glabridin could be useful for stem cell-based therapies.

## 1. Introduction

Mesenchymal stem cells (MSCs) derived from various tissues are very promising sources for cellular therapies and regenerative medicine, since they are easily accessible and differentiate into a variety of cell types, including osteoblasts, chondrocytes, and adipocytes [1, 2]. MSCs have been applied to cell-based therapies due to their numerous advantages including anti-inflammatory and immunomodulatory effects [3, 4]. However, despite the high expansion potential of MSCs, when cultured *in vitro*, they easily undergo proliferation arrest prior to significant telomere shortening due to intrinsic and/or extrinsic environmental factors [5, 6]. It is known that oxidative stress—due to an imbalance between the production of free radicals and the ability of the body to detoxify their harmful effects—is one of the main factors that induce senescence [7]. Senescence represents an arrested state in which cells remain viable but not stimulated to proliferate by serum or passage in culture. Cellular senescence of MSCs reduces functionality, which might impair their regenerative potential [8]. Therefore, MSCs need to be expanded for clinical application through *in vitro* long-term cultivation without early growth stop or cellular senescence.

Glabridin is an isoflavan compound found in the root extract of licorice [9]. A number of studies have reported that glabridin exhibits protective functions against oxidative stresses and cytotoxicity [10, 11]. In addition, it has been reported to inhibit cancer stem cell-like properties of human breast cancer cells, suggesting that it could enhance the effectiveness of breast cancer therapy [12]. Recently, it was also shown that glabridin attenuates oxidative damage and cellular dysfunction and upregulates osteoblast differentiation genes in osteoblastic cells [13]. These findings suggest the interesting possibility that glabridin could have positive effects on MSC culture *in vitro*. Although there is some evidence that glabridin protects cells from oxidative stress, no study has investigated whether glabridin can prevent MSC senescence. We therefore hypothesized that glabridin would preserve MSC functionality and would be beneficial for MSC culture *in vitro*.

The aim of this study was to examine the effects of glabridin on MSC *in vitro* culture. Treatment of MSCs with glabridin provided a favorable environment that improved stemness through upregulation of the *OCT4* gene, which is involved in pluripotency. To investigate the effects of glabridin on the differentiation potential of MSCs, we examined genes for osteogenic factors (*DLX5* and *RUNX2*), chondrogenic factors (*BMP7* and *SOX9*), and adipogenic factors (*PPARG* and *C/EBPA*). We found that the treatment of MSCs with glabridin led to osteoblast differentiation with the upregulation of osteoblast markers such as *OSTEOCALCIN* and *OSTEOPONTIN*. To our knowledge, this is the first study to indicate that glabridin is a beneficial factor that induces the *OCT4* gene and improves osteogenic differentiation of MSCs during culture *in vitro*. These findings will be useful for preparing highly functional MSCs for cell-based clinical applications.

## 2. Materials and Methods

### 2.1. Cell Culture

The bone marrow was collected from healthy donors after obtaining written informed consent. This study was approved by the Institutional Review Boards of Severance Hospital of Yonsei University Health System, Seoul, Korea. As previously described, mononuclear cells were isolated by Ficoll-Hypaque density gradient centrifugation (Pharmacia Biotech, Uppsala, Sweden), and the MSCs were cultured using the plastic adherence method [14]. The cells were maintained in DMEM Low Glucose supplemented with 10% fetal bovine serum (FBS) and 1% penicillin/streptomycin (P/S) at 37°C with 5% CO_2_ (all from Invitrogen, Carlsbad, CA, USA). The medium was changed every 3 or 4 days. The cells were subcultured using 0.05% trypsin/EDTA (Invitrogen) when they reached approximately 80–90% confluence. Glabridin (0.01–100 *μ*M) was purchased from Sigma-Aldrich (St. Louis, MO, USA).

### 2.2. Cell Viability Test

The cells were seeded into 12-well plates (Corning Inc., Corning, NY, USA) at a density of 4 × 10^4^ cells/well for assessment of cell viability. The next day, cells were treated with glabridin (0.01–100 *μ*M), directly added to the medium, for 24 h. The viability of the cells was analyzed using a CCK-8 kit (Dojindo Co., Kumamoto, Japan), which measures cell metabolic activity, according to the manufacturer's instructions [15]. Briefly, 50 *μ*M of the CCK-8 solution was added to each well of the culture plate at the end of the culture period. After 4 h of incubation, absorbance was measured at 450 nm. Cells incubated without glabridin were used as a control.

### 2.3. Cell Proliferation Assay

Cells were plated at a density of 2 × 10^4^/well in 12-well plates (Corning) for analysis of cell growth. When the cells were replated, glabridin (0.01–100 *μ*M) was added to each well of the culture plate. A proliferation assay was performed using a CCK-8 kit. CCK-8 contains WST-8 [2-(2-methoxy-4-nitrophenyl)-3-(4-nitrophenyl)-5-(2,4-dissulfophenyl)-2H-tetrazolium, monosodium salt], which produces a water-soluble formazan dye upon reduction in the presence of an electron carrier. CCK-8, being nonradioactive, allows sensitive colorimetric assays for the determination of the number of viable cells during cell proliferation. Cultures were maintained for 7 days and then analyzed for cell growth on days 1, 4, and 7 according to the manufacturer's instructions. Cells incubated with the culture medium alone were used as a control. The absorbance of the cells was normalized to their respective day 0 absorbance.

### 2.4. Reverse Transcription PCR (RT-PCR)

Total RNA was isolated using TRIzol reagent (Invitrogen). Standard reverse transcription was performed using transcriptase II (Invitrogen). RT-PCR was performed using PCR primers (Bioneer, Daejeon, Korea) under the conditions listed in Table 1. Glyceraldehyde 3-phosphate dehydrogenase (*GAPDH*) level was used as an internal control. The signal intensity of the product was normalized to its respective *GAPDH* signal intensity.

### 2.5. Colony Forming-Unit-Fibroblast (CFU-F) Assay

For assessment of self-renewal properties, a CFU-F assay was performed. Briefly, 1 × 10^3^ cells were plated in 100 mm dishes (Corning), and the cells were cultured for 14 days. Following incubation for 14 days, the cells were washed with phosphate-buffered saline (PBS; Invitrogen). Then, the cells were stained with 0.5% crystal violet (Sigma-Aldrich) for 5 min at room temperature, and stained colonies were counted.

### 2.6. *β*-Galactosidase Staining

Senescent cells show an increase in cell size and the senescence-associated expression of *β*-galactosidase activity. A senescence detection kit (BioVision Inc., CA, USA) was used to histochemically detect *β*-galactosidase activity in cultured cells, according to the manufacturer's instructions. Briefly, cultured cells were washed with PBS and fixed with 4% paraformaldehyde at room temperature. After washing with PBS, cells were incubated with *β*-galactosidase staining solution for 24 h at 37°C. The number of *β*-galactosidase-stained cells was counted under a light microscope (Olympus-IX71; Olympus, Tokyo, Japan).

### 2.7. Differentiation Assay

To induce MSC differentiation into osteoblasts, chondrocytes, and adipocytes, cells were cultured in osteogenic induction medium, chondrogenic induction medium, or adipogenic induction medium for 3 weeks (Cambrex, Lonza, MD, USA), respectively. The medium was changed every 3 or 4 days, and the cells for chondrogenic differentiation were treated with 10 ng/ml transforming growth factor (TGF)-*β*3 (Cambrex) whenever the medium was replaced. The induced cells were stained with von Kossa to confirm osteogenesis, safranin O to confirm chondrogenesis, and oil red O to confirm adipogenesis. Images of the stained cells were taken using a phase microscope (Olympus-IX-71). To measure the calcium content in osteogenesis, the Calcium LiquiColor kit (Stanbio Laboratory, Boerne, USA) was used according to a previously described method [16]. Briefly, the cells were washed with PBS and treated with 0.5 N HCl. After shaking for 3 h by using an orbital shaker, the supernatant was transferred to a new tube for analysis. Ortho-cresolphthalein complexone (OCPC) was added to the sample, and absorbance was determined at 550 nm. For quantitative analysis of adipogenesis, absorbance was measured at 500 nm after destaining with isopropanol for 30 min according to a previously reported method [16]. To quantitatively evaluate chondrogenesis, the absorbance of sulfated glycosaminoglycan was measured at 656 nm by using the Blyscan assay kit (Biocolor Ltd., County Antrim, UK). Briefly, the supernatant was transferred to a new tube and each sample was mixed with 1,9-dimethylmethylene blue (DMMB) dye, which is used to measure sulfated glycosaminoglycan (sGAG) content, according to the manufacturer's instructions and a previous report [17].

### 2.8. Statistical Analysis

Quantitative data are expressed as the means ± standard deviation (SD). Statistical comparisons were performed by a Student's *t*-test and one-way analysis of variance (ANOVA) with post hoc Bonferroni corrections. The differences were considered statistically significant at *P* < 0.05. Statistical analyses were performed using SPSS software (SPSS Inc., Chicago, IL, USA).

## 3. Results

### 3.1. Effect of Glabridin on the Viability and Proliferation of MSCs

**Figure 1:**
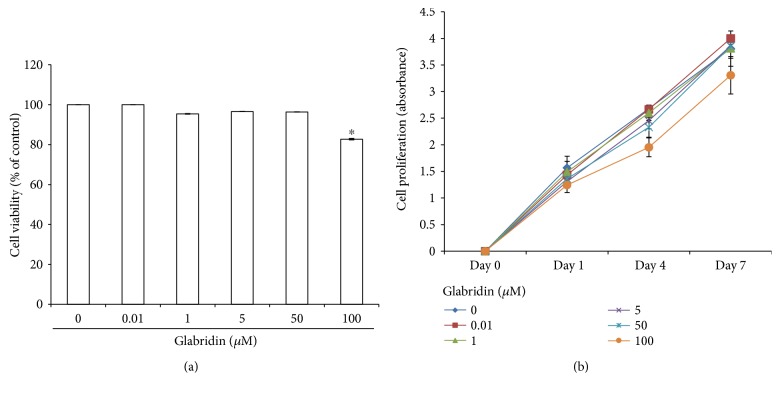
(a) Effect of glabridin on cell viability in bone marrow-derived mesenchymal stem cells (MSCs). Cell viability of MSCs treated with an increasing concentration of glabridin was determined by a CCK-8 assay. The data are expressed as the mean ± SD of three independent experiments. ^∗^*P* < 0.05 versus untreated control. (b) Growth rates of cultured MSCs. Cells were cultivated with an increasing concentration of glabridin for 7 days. Proliferation activity was measured using a CCK-8 kit containing WST-8. The data are expressed as the mean ± SD of three independent experiments.

To investigate the effect of glabridin on MSC survival, cells were cultured with increasing concentrations (0.01–100 *μ*M) of glabridin for 24 h and then cell viability was measured using a CCK-8 assay. Glabridin at concentrations of 0.01–50 *μ*M had no effect on cell survival, whereas incubation with 100 *μ*M glabridin decreased the cell viability of MSCs (Figure 1(a)). These results show that 100 *μ*M glabridin itself was cytotoxic to MSCs.

To evaluate the effect of glabridin on MSC proliferation, MSCs were cultured for 7 days to determine whether glabridin stimulates MSC growth, and then cell proliferation was determined using the CCK-8 assay. A decrease in cell growth was detected in MSCs cultured with 100 *μ*M glabridin, while the cells cultured with 0.01–50 *μ*M glabridin exhibited proliferation properties similar to those of the control cells (Figure 1(b)). These results show that glabridin did not facilitate the growth rates of the MSCs.

### 3.2. Effect of Glabridin on MSC Stemness

**Figure 2:**
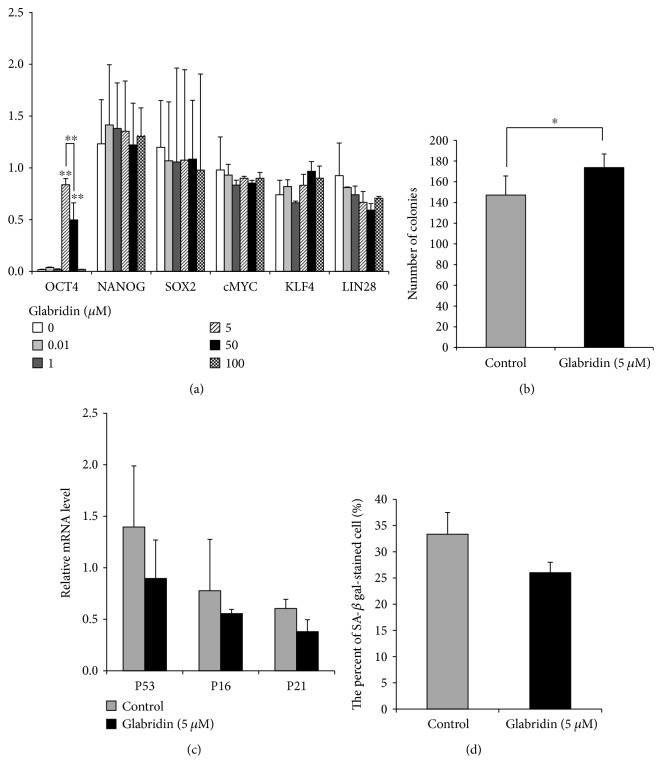
Effect of glabridin on stemness and senescence in MSCs. (a) Stemness marker expression in MSCs treated with an increasing concentration of glabridin. (b) Stemness was evaluated by a CFU-F assay. The number of colonies (>50 cells) was counted. (c) *P53*, *P16*, and *P21* mRNA expression levels were analyzed using reverse transcription PCR (RT-PCR). (d) Senescence-associated (SA) *β*-gal assay. The number of *β*-gal-positive cells was counted. The data are expressed as the mean ± SD of three experiments. ^∗^*P* < 0.05 and ^∗∗^*P* < 0.01 versus untreated control.

In order to identify the self-renewal capacity of MSCs, we analyzed the expression levels of stemness markers in the MSCs cultured with 0.01–100 *μ*M glabridin. *NANOG*, *SOX2*, *cMYC*, *KLF4*, and *LIN28* were similarly expressed with all concentrations of glabridin (Figure 2(a)). Interestingly, the *OCT4* gene—involved in the self-renewal of undifferentiated stem cells—was markedly detected in MSCs cultured with 5 *μ*M glabridin (Figure 2(a)). Therefore, we chose this concentration of glabridin (5 *μ*M) for all subsequent cell experiments. The CFU-F assay was used to investigate whether *OCT4* expression with glabridin promotes the self-renewal capacity of MSCs. Glabridin significantly enhanced the self-renewal capacity of the treated cells compared with that of the control cells (Figure 2(b)). In addition, to confirm whether glabridin affected the cell cycle, the mRNA expression levels of *P53*, *P16^INK4a^*, and *P21^Cip1^* in MSCs treated with glabridin were measured. The expression levels of *P53*, *P16^INK4a^*, and *P21^Cip1^* of MSCs treated with glabridin were decreased compared to those in control cells (Figure 2(c)). We next examined whether glabridin prevented MSC senescence. MSCs treated with glabridin showed a decrease in the percentage of *β*-galactosidase-stained cells compared to control cells although there was no significance (Figure 2(d)). Together, these results suggest that glabridin augments the self-renewal capacity with upregulation of the *OCT4* gene.

### 3.3. Changes of Differentiation Potential in MSCs by Glabridin

**Figure 3:**
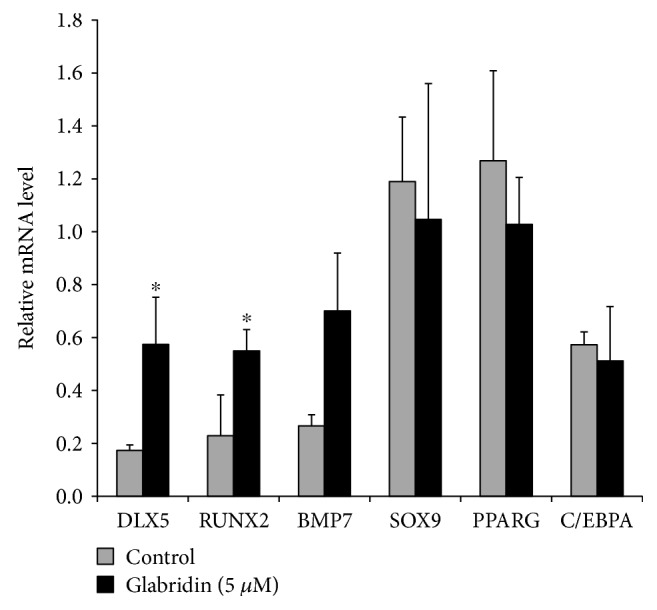
Gene expression in MSCs following glabridin treatment. RT-PCR analysis of osteogenic, chondrogenic, and adipogenic markers was performed in control and glabridin-treated MSCs. Relative mRNA expression levels of trilineage-associated genes in the control and glabridin-treated MSCs. Expression levels relative to *GAPDH* are shown. The data are expressed as the mean ± SD of three experiments. ^∗^*P* < 0.05 versus untreated control.

We subsequently examined the osteogenesis-, chondrogenesis-, and adipogenesis-related gene expression levels in the MSCs after glabridin treatment. *DLX5* and *RUNX2* genes, which are involved in osteogenesis, were markedly upregulated by glabridin (Figure 3). With respect to chondrogenesis, the *BMP7* gene was upregulated, whereas the expression of *SOX9* gene was similar to MSCs treated with glabridin (Figure 3). The adipogenesis-related *C/EBPA* gene was similarly expressed in glabridin-treated MSCs compared to that in control cells, while the expression level of the *PPARG* gene decreased with glabridin treatment (Figure 3). These results indicate that glabridin strongly affects the osteogenic potential of MSCs.

### 3.4. Glabridin Enhances Osteogenesis of MSCs

**Figure 4:**
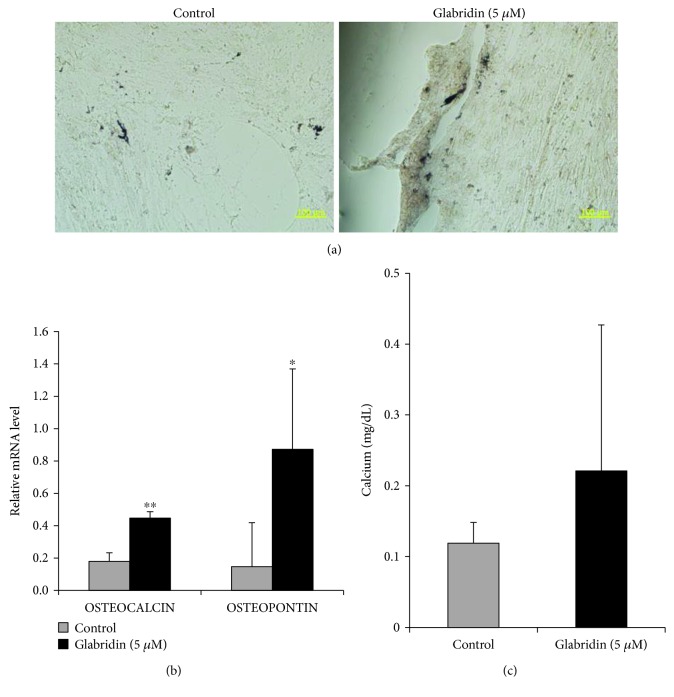
Effect of glabridin on osteogenesis in MSCs. (a) Osteogenic differentiation was evaluated by von Kossa staining (magnification: 200x). (b) Osteogenic potential was analyzed by *OSTEOCALCIN* and *OSTEOPONTIN* gene expression using RT-PCR. (c) Differentiation of MSCs into osteoblasts was determined by calcium quantification. The data are expressed as the mean ± SD of three experiments. ^∗^*P* < 0.05 and ^∗∗^*P* < 0.01 versus untreated control.

**Figure 5:**
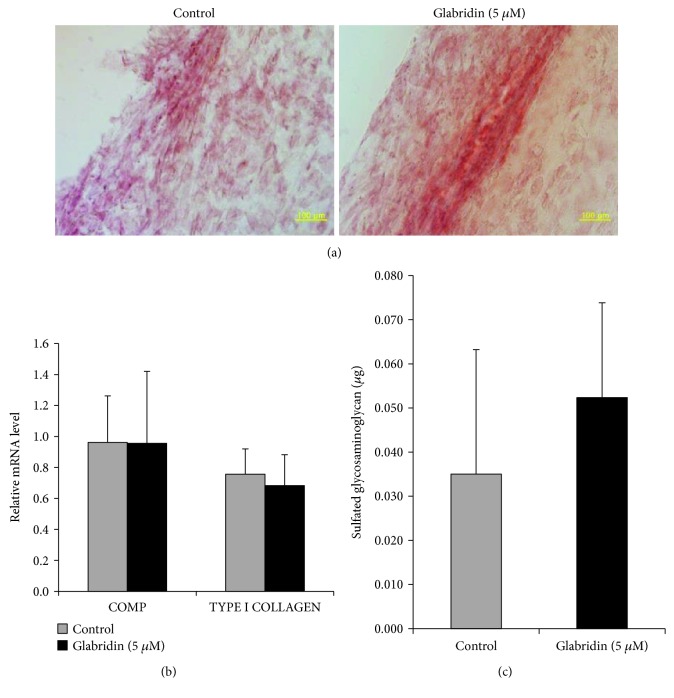
Effect of glabridin on chondrogenesis in MSCs. (a) Chondrogenic differentiation was evaluated by safranin O staining (magnification: 200x). (b) Chondrogenic potential was analyzed by levels of *COMP* and *TYPE I COLLAGEN* gene expression using RT-PCR. (c) Differentiation of MSCs into chondrocytes was determined by glycosaminoglycan quantification. The data are expressed as the mean ± SD of three experiments.

**Figure 6:**
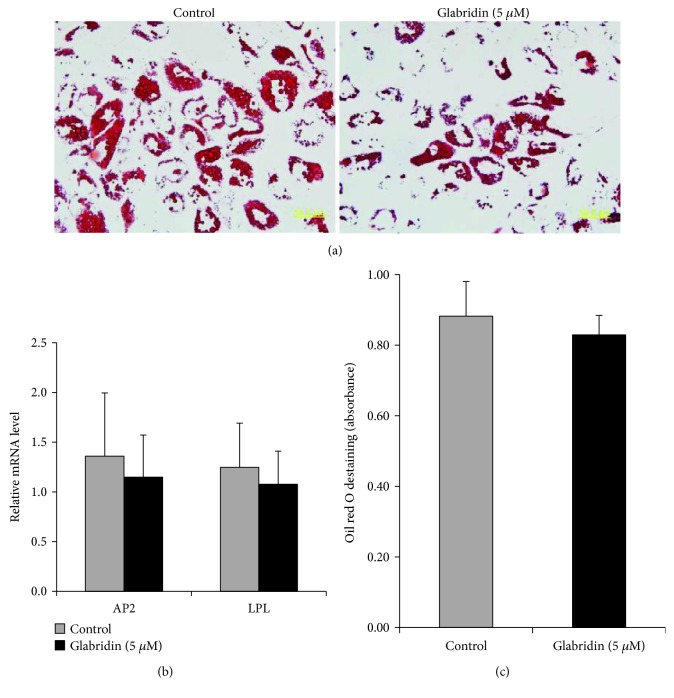
Effect of glabridin on adipogenesis in MSCs. (a) Adipogenic differentiation was evaluated by oil red O staining (magnification: 400x). (b) Adipogenic capacity was analyzed by *AP2* and *LPL* mRNA expression using RT-PCR. (c) Absorbance was determined after oil red O destaining for quantitative analysis. The data are expressed as the mean ± SD of three experiments.

To evaluate the differentiation capacity of MSCs, cells were induced by glabridin to form osteoblasts, chondrocytes, and adipocytes. MSCs treated with glabridin showed higher amounts of von Kossa staining, which detects calcium-containing mineralized nodules, compared to control cells (Figure 4(a)), and the expression of *OSTEOCALCIN* and *OSTEOPONTIN* genes of osteogenic differentiation markers was upregulated in MSCs treated with glabridin (Figure 4(b)). Moreover, we confirmed that MSCs cultured with glabridin had a higher degree of calcium accumulation compared to control cells although there were no significant differences (Figure 4(c)). Chondrogenesis was assessed by safranin O staining. After chondrogenic induction, MSCs cultured with glabridin exhibited a slightly higher chondrogenic differentiation capacity (Figure 5(a)). However, the *COMP* and the *TYPE I COLLAGEN* genes of the chondrogenic differentiation markers were similarly expressed despite chondrogenic induction in both conditions (Figure 5(b)). Sulfated glycosaminoglycan content was slightly increased in MSCs grown with glabridin, irrespective of the PCR data (Figure 5(c)). There were no significant differences. In adipogenesis, glabridin slightly suppressed adipogenic differentiation as shown in Figure 6(a). In addition, the *AP2* and *LPL* genes of adipogenesis-related markers were slightly decreased in glabridin-treated cells without a significant difference (Figure 6(b)). We also confirmed that the absorbance value of lipid droplets was reduced in MSCs induced with glabridin (Figure 6(c)). Together, these results imply that glabridin prominently enhanced the osteogenic differentiation capacity of MSCs by upregulating the *OSTEOCALCIN* and *OSTEOPONTIN* genes.

## 4. Discussion

Of the adult stem cells, MSCs have been widely used for clinical applications because of their plastic and anti-inflammatory effects [18, 19]. Although MSCs represent a new approach to treat intractable diseases, clinical trials using these cells have been impeded by low quantities of cells and difficulty of cell culture, described as cellular or replicative senescence. MSCs can easily enter a state of growth arrest, known as senescence, despite high self-renewal capacity by internal and/or external stimuli [5]. Therefore, the culture and maintenance of MSCs without the loss of stemness are very critical for their extensive clinical use.

In general, cells are affected by multiple biochemical and biophysical factors such as the extracellular matrix (ECM) and soluble factors [20, 21]. Previously, we prevented senescence and augmented MSC growth using poly-L-lysine (PLL) of ECM proteins [22]. PLL definitely improved the proliferation capacity and functionality of MSCs. However, using PLL as a coating substrate is time-consuming because culture vessels should be incubated and dried for a long time after PLL coating. Recently, Kim et al. reported that glabridin, one of the major active flavonoids in licorice, attenuates oxidative damage and improves osteogenic differentiation function [13]. It was also reported that glabridin inhibits the cancer stem cell-like properties in hepatocellular carcinoma cells [23]. Cellular senescence is very closely related to oxidative stress [24]. In the present study, we applied glabridin, which has an antioxidant activity, to a cell culture condition. We found that glabridin did not significantly affect the viability of bone marrow-derived MSCs. In addition, glabridin did not activate cell proliferation in *in vitro* culture. We analyzed the expression levels of *OCT4*, *NANOG*, *SOX2*, *cMYC*, *KLF4*, and *LIN28* genes known as stemness markers of stem cells to investigate molecular patterns of self-renewal capacity. *NANOG*, *SOX2*, *cMYC*, *KLF4*, and *LIN28*, which are involved in pluripotency and self-renewal of stem cells, were similarly expressed in all conditions. *OCT4*, an essential transcription factor in the maintenance of pluripotency, is expressed in embryonic stem cells [25]. In addition, *OCT4* is a very important gene for the generation of induced pluripotent stem cells [26]. It is known that *OCT4*, as a specific marker of embryonic stem cells, is also expressed in MSCs [27]. However, it is hard to detect the expression of *OCT4* in MSCs because it readily disappears during culture *in vitro* [28]. Recently, Piccinato et al. showed that a high *OCT4* gene expression might be a potential hallmark and predictor of greater *in vitro* lifespan and growth potential of MSCs [29]. These results indicate that the expression level of the *OCT4* gene may be a specific factor that affects MSC senescence.

In this study, the *OCT4* gene was strongly induced in the presence of 5 *μ*M glabridin. However, there was no dose-dependent *OCT4* expression by glabridin. Rather, MSCs treated with 100 *μ*M glabridin did not express *OCT4*. The expression of *OCT4* might be suppressed by a decrease in viability as shown in Figure 1. When MSCs were treated with glabridin, a significant increase in their self-renewal capacity was observed, implying that glabridin enhances CFU-F capacity by inducing the *OCT4* gene. Several studies have reported that cellular senescence is regulated by the *P53*, *P16^INK4a^*, and *P21^Cip1^* pathways via the accumulation of reactive oxygen species [30–32]. We have confirmed that the *P53*, *P16^INK4a^*, and *P21^Cip1^* mRNA expression levels were inhibited by glabridin although there was no significance. These results coincide with previous results, demonstrating that cellular senescence of MSCs could be suppressed via inhibition of *P53* and *P21^Cip1^* [33]. In addition, glabridin delayed MSC senescence as observed by *β*-galactosidase staining, showing that glabridin positively affects cell senescence in MSCs cultured *in vitro*. Together, these results strongly support that glabridin has antisenescence effects.

Recently, it was reported that MSC treatment with glabridin resulted in a significant elevation of alkaline phosphatase (ALP) activity, collagen content, and expression of osteoblast differentiation genes [13]. To investigate the effects of glabridin on MSC differentiation potential, trilineage (osteogenesis, chondrogenesis, adipogenesis)-related key transcription factors were analyzed by RT-PCR after treatment with glabridin as compared with control cells. When MSCs were treated with glabridin, significant increases in the gene expression of *DLX5* and *RUNX2* for osteogenesis were observed. In the differentiation assay, our results demonstrated that glabridin could significantly improve osteogenic differentiation capacity with significant upregulation of *OSTEOCALCIN* and *OSTEOPONTIN* genes of osteogenesis markers. Regarding chondrogenesis, sulfated glycosaminoglycan contents were also elevated by glabridin, but glabridin had no effects on the chondrogenic differentiation marker genes. Moreover, glabridin slightly attenuated adipogenic differentiation capacity with changes in the expression levels of genes associated with adipogenesis. These results correspond to a previous result, demonstrating that overexpression of *DLX5*, a key factor for osteogenesis, inhibited the expression of adipogenic marker genes [34]. In the present results, enhancement of osteogenesis inhibited adipogenesis of human MSCs, due to the reverse relationship between osteogenic and adipogenic differentiation.

**Figure 7:**
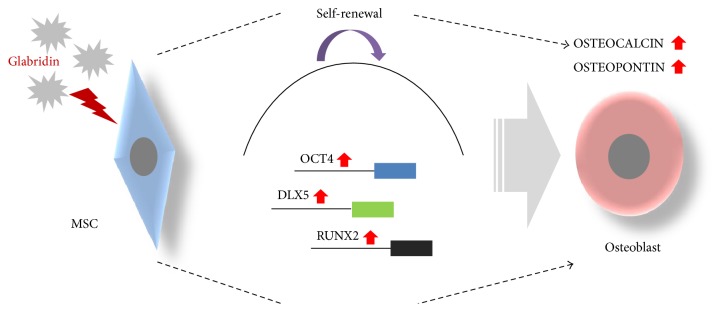
Schematic summary of the effect of glabridin on osteogenesis of MSCs. Our results suggest that glabridin upregulates the expression level of the *OCT4* gene associated with stemness and that of *DLX5* and *RUNX2* genes related to osteogenesis. Glabridin enhances *OCT4*-induced osteogenesis of MSCs by activating *OSTEOCALCIN* and *OSTEOPONTIN* genes for osteogenic differentiation.

In summary, we have shown that glabridin improved osteogenic differentiation capacity of MSCs by inducing the expression of the *OCT4* gene of the pluripotency factors and augmenting *DLX5* and *RUNX2* gene expression for osteogenesis as shown in Figure 7. We thus conclude that glabridin could be used in the MSC culture system *in vitro*. Furthermore, MSC culture using glabridin will contribute greatly to regenerative medicine and cell-based therapies including bone diseases.

## Figures and Tables

**Table 1:** Primer sequences used for RT-PCR.

Gene	Primer sequence (5′–3′)	Annealing temperature (°C)	Product size (bp)
*GAPDH*	Forward: GTGGTCTCCTCTGACTTCAACA Reverse: CTCTTCCTCTTGTGCTCTTGCT	62	210
*OCT4*	Forward: GACAACAATGAGAACCTTCAGGAGA Reverse: TTCTGGCGCCGGTTACAGAACCA	62	218
*SOX2*	Forward: AACCAAGACGCTCATGAAGAAG Reverse: GCGAGTAGGACATGCTGTAGGT	62	341
*cMYC*	Forward: TCGGATTCTCTGCTCTCCTC Reverse: CGCCTCTTGACATTCTCCTC	62	413
*KLF4*	Forward: ATTCTCTCCAATTCGCTGACCC Reverse: TTCAGCACGAACTTGCCCAT	62	376
*NANOG*	Forward: ATAGCAATGGTGTGACGCAG Reverse: GATTGTTCCAGGATTGGGTG	62	219
P53	Forward: TCGACATAGTGTGGTGGTGC Reverse: TTGGACTTCAGGTGGCTGGA	58	480
*LIN28*	Forward: GCTCCGTGTCCAACCAGCAG Reverse: TTTCCTTTTGGCCGCCTCTC	58	376
P16	Forward: CGAATAGTTACGGTCGGAGG Reverse: GCATGGTTACTGCCTCTGGT	62	309
*DLX5*	Forward: ACCATCCGTCTCAGGAATCG Reverse: ACCTTCTCTGTAATGCGGCC	60	384
*RUNX2*	Forward: TTGCAGCCATAAGAGGGTAG Reverse: GTCACTTTCTTGGAGCAGGA	58	470
*PPARG*	Forward: TCTCTCCGTAATGGAAGACC Reverse: GCATTATGAGACATCCCCAC	55	474
*C/EBPA*	Forward: CCAAGAAGTCGGTGGACAAGAA Reverse: TCATTGTCACTGGTCAGCTCCA	62	145
*BMP7*	Forward: CCAACGTCATCCTGAAGAAATAC Reverse: GCTTGTAGGATCTTGTTCATTGG	60	271
*SOX9*	Forward: GGTTGTTGGAGCTTTCCTCA Reverse: TAGCCTCCCTCACTCCAAGA	61	400
P21	Forward: GCGATGGAACTTCGACTTTG Reverse: CGTTTTCGACCCTGAGAGAGTC	60	285
*OSTEOCALCIN*	Forward: CGCAGCCACCGAGACACCAT Reverse: GGGCAAGGGCAAGGGGAAGA	62	405
*OSTEOPONTIN*	Forward: GAGACCCTTCCAAGTAAGTCCA Reverse: GATGTCCTCGTCTGTAGCATCA	62	354
*COMP*	Forward: GAAGAACGACGACCAAAAGGAC Reverse: GTCACAAGCATCTCCCACAAAG	62	232
*TYPE I COLLAGEN*	Forward: CACAGAGGTTTCAGTGGTTTGG Reverse: GCACCAGTAGCACCATCATTTC	62	191
*AP2*	Forward: AAGAAGTAGGAGTGGGCTTTGC Reverse: CCACCACCAGTTTATCATCCTC	62	381
*LPL*	Forward: AGAGAGGACTTGGAGATGTGGA Reverse: GGAAGACTTTGTAGGGCATCTG	62	264
